# Anatomical Considerations for Gallbladder Sonography: A Cross-Sectional Study of CT Imaging by BMI Group

**DOI:** 10.7759/cureus.40557

**Published:** 2023-06-17

**Authors:** Sophie A Halpern, Harrison A Patrizio, Eamonn J Brace, Shiyuan Wang, Jonathan Y Yuh, Dip V Patel, Ryan G Morrison, Arielle J Hall, Amerigo Falciani, Kathleen A Deiling, Nils V Brolis

**Affiliations:** 1 Clinical Education and Assessment Center, Rowan-Virtua School of Osteopathic Medicine, Stratford, USA

**Keywords:** hepatobiliary, medical education, obesity, ultrasound, gallbladder

## Abstract

Objective

The purpose of this study was to establish an association between the body mass index (BMI) group and anatomical gallbladder position to aid novices in gallbladder sonography.

Methods

This was a cross-sectional, Strengthening the Reporting of Observational Studies in Epidemiology (STROBE)-compliant study that examined the association between gender and the BMI group with quantitative gallbladder position measurements from computed tomography (CT) scans.

Results

A quantitative analysis determined that the gallbladder was positioned relatively higher and oriented more horizontally within the abdomen of individuals with obese BMI than those with normal BMI (p < 0.001), irrespective of gender. Additionally, the gallbladder was more obstructed by the rib cage in individuals with obese BMI than those with normal BMI (p = 0.007 for females and p < 0.001 for males). The gallbladder was significantly more horizontal in overweight males than females (p < 0.001) and more obstructed by the rib cage in obese males than females (p = 0.013).

Conclusion

This association provides ultrasound novices knowledge for a more targeted approach in localizing the gallbladder and evidence to recommend an intercostal approach for gallbladder sonography in obese patients.

## Introduction

Gallbladder sonography is a challenging diagnostic procedure with one of the highest error rates in radiology [1]. These errors are likely due to fluctuations in gallbladder size and the organ’s deep intraperitoneal location with proximity to the liver at its superior surface [2]. The ultrasound of the gallbladder can be especially difficult in individuals with increased body mass index (BMI), which can alter image quality and accuracy [3]. In theory, the gallbladder should be in a similar position relative to anatomical landmarks based on an individual’s body shape and size. Despite this, the gallbladder can be found at various locations within the abdomen, both well above or below the right costal margin [4].

The positioning of abdominal organs may be influenced by gender, BMI, and muscle composition [5]. The determinants of anatomical gallbladder position are not widely known nor referenced in protocols for gallbladder ultrasound [6]. The purpose of this cross-sectional study was to establish an association between BMI and anatomical gallbladder position within the abdomen so that protocols for gallbladder sonography can be better specified.

## Materials and methods

Study design

This was a cross-sectional study that collected patients’ de-identified demographics and quantitative gallbladder position measurements for analysis. Data was collected at one point in time from previous records. The gallbladder position measurements were collected using non-contrast computed tomography (CT) imaging instead of ultrasound for the repeatability of the measurements between patients. This study was completed in compliance with the Strengthening the Reporting of Observational Studies in Epidemiology (STROBE) checklist and approved by the Rowan University Institutional Review Board (IRB) (IRB number: PRO-2022-57) [7]. Some study data was previously presented at the 2023 Rowan-Virtua School of Osteopathic Medicine Annual Research Day on May 4, 2023.

Setting

Data was collected remotely through retrospective chart review using Fujifilm’s Synapse (Tokyo, Japan) electronic medical record (EMR) and picture archiving and communication system (PACS). All CT scans were performed at 10 different locations within the same private radiology practice between April 2018 and April 2019. All locations utilized a General Electric (GE) Lightspeed 16/64-Slice CT machine.

Participants

Patients aged 18-40 years old with varying BMIs and a physiologically distended gallbladder visible on CT were selected for analysis. Several inclusion and exclusion criteria helped narrow down this patient population. CT scans were selected for analysis based on the following inclusion criteria: The purpose of the scan was for suspected urolithiasis; the age of the patient was between 18 and 40 years old at the time the scan was performed; the chart including the scan contained the record of the patient’s age, gender, height, and weight; and CT scan revealed a visible, physiologically distended gallbladder. Suspected urolithiasis was the chosen pathology to narrow the patient population due to its lack of related pathophysiology to the hepatobiliary system, its likelihood of occurrence in the desired patient population age range, and the convenience of having its own categorical designation within the PACS system. The age range of 18-40 years old was chosen to avoid a possible confounding variable of age-related liver size changes [8].

CT scans were then excluded for the following findings: The gallbladder was contracted or absent, gallbladder pathology was present, liver pathology was present, and umbilicus, xiphoid process, or 10th rib was not clearly identifiable. CT scans with gallbladder and liver pathology were excluded to eliminate possible confounding variables that could alter the anatomical position of the gallbladder. CT scans were only included if the gallbladder was physiologically distended so that measurements were taken in a state optimized for gallbladder sonography.

Variables

**Figure 1:**
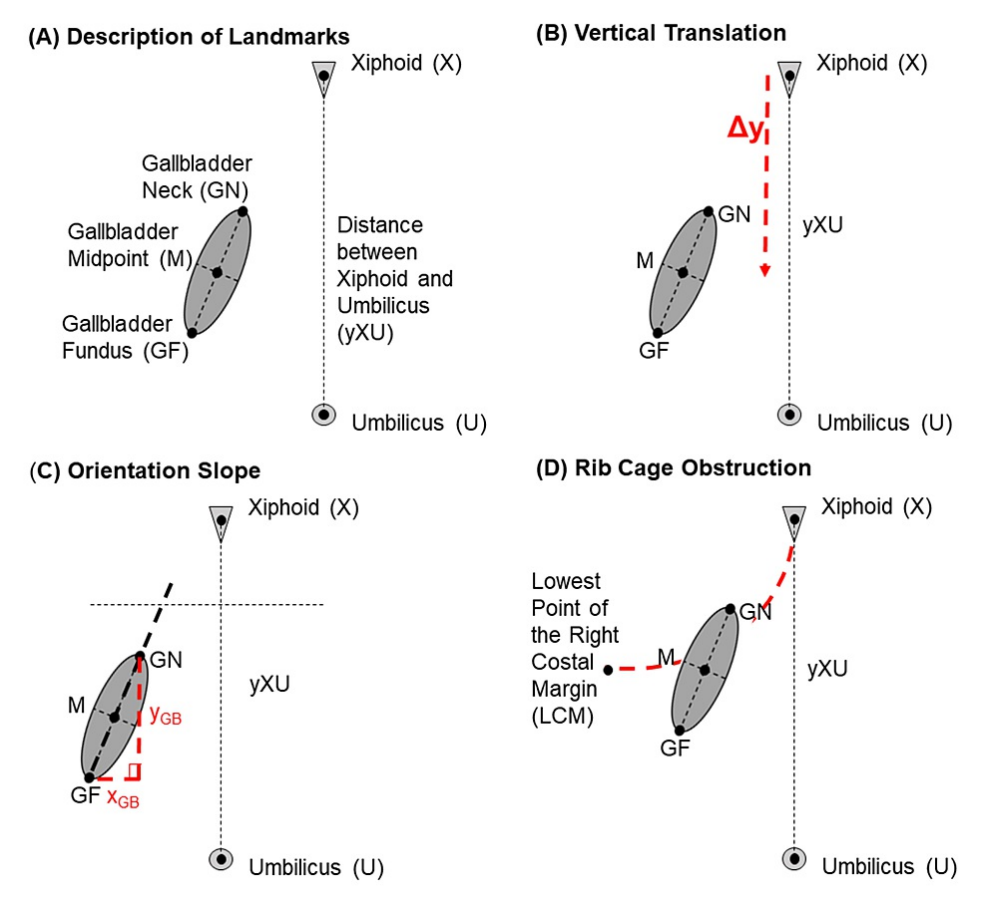
Description of Anatomical Landmarks

**Figure 2:**
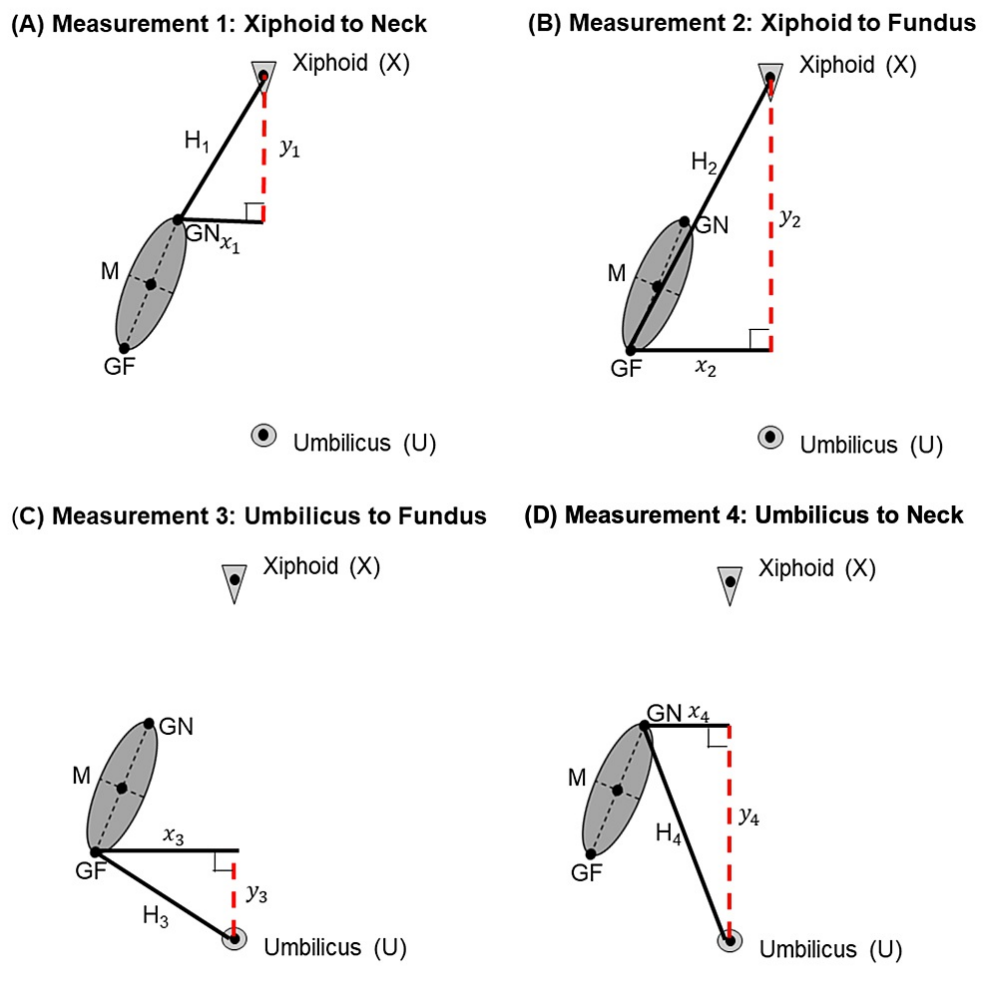
Visualization of Linear Measurements in the Coronal Plane GF, gallbladder fundus; M, gallbladder midpoint; GN, gallbladder neck

**Figure 3:**
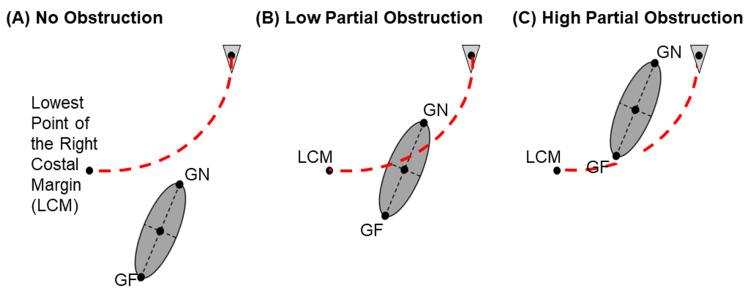
Visualization of Likert Scale Model for Rib Cage Obstruction GF, gallbladder fundus; GN, gallbladder neck

The independent variables of this study were gender and the BMI group. The BMI group was designated by defined groups from the Centers for Disease Control and Prevention [9]. Three dependent variables were obtained to define the anatomical position of the gallbladder within the abdomen relative to other landmarks (Figure 1A): (1) The relative vertical translation of the gallbladder was calculated on a one-dimensional y-axis defined by the distance between the xiphoid process and umbilicus (Figure 1B), (2) the orientation of the gallbladder was calculated as the slope of the line drawn between the gallbladder neck and fundus to understand if the organ was in a more horizontal or vertical position (Figure 1C), and (3) obstruction by the rib cage was represented by a Likert scale model to rate the degree to which the gallbladder was obstructed by the rib cage (Figure 1D).

Data measurements

The dependent variables were calculated based on linear measurements drawn via the Fujifilm PACS software. The technique for the linear measurements between landmarks was modified from established techniques for other abdominal organs [10,11]. The following landmarks were established with agreement across the sagittal, coronal, and transverse planes of measurement in abdominal CT: the xiphoid process, umbilicus, neck of the gallbladder, and fundus of the gallbladder. The neck was defined as the point of the gallbladder most proximal to the biliary tree at the liver hilum. The fundus was defined as the most distal point of the gallbladder. Two-dimensional linear measurements were made between the gallbladder neck and either the estimated midpoint of the xiphoid or umbilicus. This was also done between the gallbladder fundus and either the estimated midpoint of the xiphoid or umbilicus. Each individual measurement was taken in a single plane. These measurements were conducted twice using the coronal and sagittal views separately (Figure 2).

Data analysis

Vertical translation from the xiphoid process was calculated as a ratio relative to the total distance between the xiphoid and umbilicus (Appendices). This was done to correct for variations in distance due to different patient heights. A larger ratio (approaching one) of relative vertical translation suggests that the gallbladder is positioned relatively lower in the abdomen and closer to the umbilicus than the xiphoid. A smaller ratio (approaching zero) of relative vertical translation suggests that the gallbladder is positioned relatively higher in the abdomen and closer to the xiphoid than the umbilicus. For orientation, a straight line was drawn through the neck and fundus in the coronal or sagittal plane. The slope of this line was calculated in the coronal and sagittal planes to determine the orientation of the gallbladder in both dimensions (Appendices). A larger slope value indicates that the gallbladder is in a more vertical orientation. A smaller slope value indicates that the gallbladder is in a more horizontal position. Because the range of slope is from zero to infinity, a log transformation was calculated with the slope values to achieve a normal distribution for statistical analysis.

Obstruction by the rib cage was measured with a Likert scale model signifying the degree to which the gallbladder was obstructed by the rib cage at the lowest point of the right costal margin (LCM) at the midaxillary line (Figure 3). A score of “0” was recorded if both the gallbladder neck and gallbladder fundus were not vertically positioned higher than the LCM at the right midaxillary line. This score implied that there was no obstruction of the gallbladder by the rib cage when viewing the gallbladder coronally or sagittally. A score of “1” was recorded if the gallbladder neck was vertically positioned higher than the LCM but the gallbladder fundus was not. This score implied that there was a partial obstruction of the gallbladder by the rib cage when viewing the gallbladder coronally or sagittally. A score of “2” was recorded if both the gallbladder neck and gallbladder fundus were vertically positioned higher than the LCM at the right midaxillary line. This score implied that there was likely a total obstruction of the gallbladder by the rib cage when viewing the gallbladder sagittally and possible partial obstruction when viewing the gallbladder coronally. All graphics were created via Microsoft PowerPoint (Microsoft® Corp., Redmond, WA) and Fusion 360 (Autodesk, San Francisco, CA).

Measurement reliability and bias

Two separate data collectors were responsible for collecting the independent and dependent variables separately so that the collector performing the linear measurements from the CT scan was unaware of the patient’s numerical BMI. To establish the reliability of the three dependent variables, vertical translation, orientation, and obstruction to the rib cage (the Likert scale), another two data collectors independently collected the data of the three dependent variables from 18 CT scans (10% of the total sample size), randomly drawn from the sample and balanced by gender and BMI groups. A single measure of two-way mixed-effect interclass correlation (ICC) model for absolute agreement was used (16) to establish reliabilities on all individual linear measurements that were used to calculate each of the three dependent variables. This test revealed that the three data collectors reached good to excellent agreement on the measurement of the 16 individual vectors drawn on the CT images (ICC range, 0.82-0.99; 95% CI, 0.81-0.99), vertical translation (ICC, 0.90; 95% CI, 0.81-0.96), coronal log(slope) (ICC, 0.87; 95% CI, 0.74-0.94), sagittal log(slope) (ICC, 0.75; 95% CI, 0.54-0.88), and obstruction to the rib cage (ICC: 1, perfect agreement).

Study size

The sample size of this study was a total of 180 patients with 30 patients in each BMI and gender subgroup: (1) normal-weight females, (2) normal-weight males, (3) overweight females, (4) overweight males, (5) obese females, and (6) obese males. This sample size was arrived at through a power analysis of a small pilot trial of 60 total patients. The final sample size was determined to make sure both the overall comparison of the group difference and all pairwise comparisons of subgroups reach at least a power of 0.8. The data from the pilot study yielded significant overall and only some pairwise comparisons (p < 0.05) achieved a power of 0.8 from the sample of 60, so the sample size was increased to 30 patients per group to achieve a normal distribution and balanced sample size within each group. The first 180 patients that met the inclusion criteria based on the goal of obtaining 30 patients per BMI and gender group were selected. The selection process ceased once these goals were achieved.

Statistical methods

It was hypothesized that the dependent variables may differ by both BMI groups and gender, and the differences between BMI groups may be affected by gender. One-way analyses of variance (ANOVAs) were used to compare the six groups on vertical translation and the log transformation of coronal and sagittal plane slopes. Pearson’s correlation was also used to establish the relationship of orientation on the coronal and sagittal planes. A P-value lesser than 0.05 was used as the level of marginal significance for the vertical translation, orientation analyses, and Pearson’s correlation. All pairwise comparisons were conducted using Tukey honestly significant difference (HSD) tests except for the pairwise comparisons of vertical translation. Tamhane’s tests were used because Levene’s test suggested the heterogeneity of variance.

A Kruskal-Wallis one-way analysis of variance was performed to determine if there was a statistically significant difference between the Likert scale rib cage obstruction scores in females categorized by normal, overweight, and obese BMIs. This was repeated for males. Then, multiple Mann-Whitney U tests were run to determine which female BMI group accounted for the Kruskal-Wallis test result. Each female BMI group was compared with the other two female BMI groups. This was again repeated for males. Finally, three Mann-Whitney U tests were run to determine if there was a statistically significant difference between males and females in the normal, overweight, and obese BMI groups. To account for the three different comparisons, a Bonferroni correction was used to establish a more conservative P-value of 0.05/3 or 0.0167 for the Likert scale analysis.

## Results

Participants

**Figure 4:**
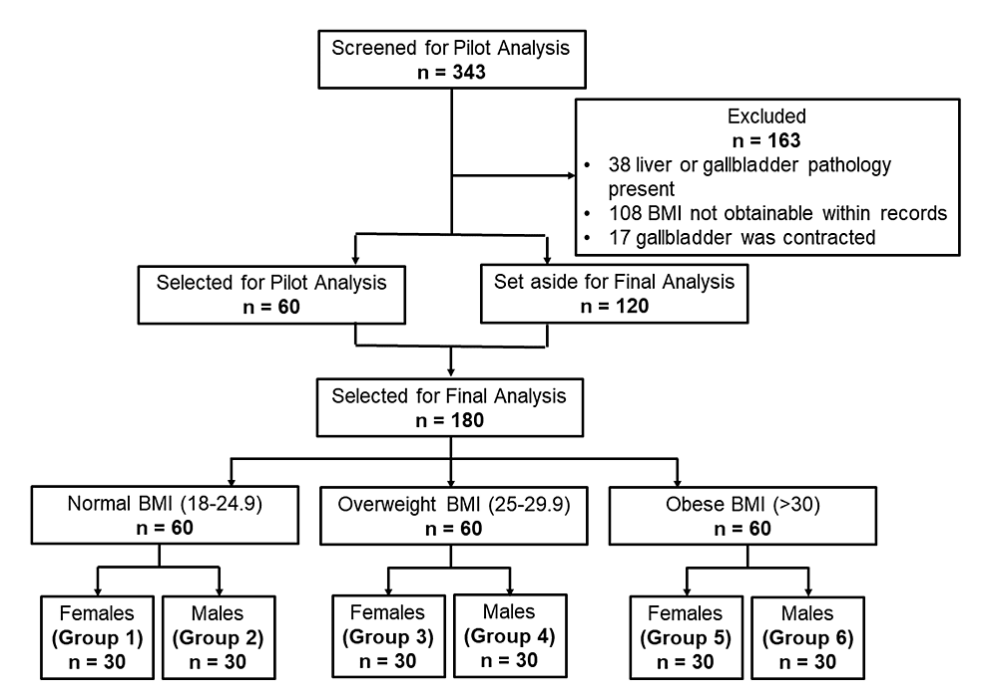
Participant Selection and Allocation for Cross-Sectional Analysis BMI: body mass index

The participants were yielded from a search of non-contrast CT scan urolithiasis studies with filtering features for patient age range and study date range. Three hundred forty-three studies were yielded to be assessed for eligibility with the goal of a sample size of 180 (Figure 4). Thirty-eight studies were excluded for the presence of gallbladder or liver pathology including hepatic steatosis/hepatomegaly (19), previous cholecystectomy (15), gallstones (3), and diffuse gallbladder wall thickening (1). The minimum, maximum, and average BMI values were recorded for each group (Table 1).

**Table 1:** BMI Demographics by Gender and Group BMI: body mass index

BMI	Female			Male		
	Normal	Overweight	Obese	Normal	Overweight	Obese
Minimum	18.66	25.09	30.11	18.65	25.02	30.26
Maximum	24.43	29.53	40.69	24.89	29.89	36.61
Average	22.22	27.11	34.53	21.92	27.18	33.15

Vertical translation

Relative vertical translation was calculated for the six groups to determine if the gallbladder was positioned higher in the abdomen and closer to the xiphoid (value → 0) or lower in the abdomen and closer to the umbilicus (value → 1) (Table 2). Statistically, one-way ANOVA of relative vertical translation values (p < 0.001) showed that there were overall significant differences between the six groups. The gallbladders of the obese BMI groups were positioned significantly higher in the abdomen than those of the respective gender and normal BMI group (p < 0.001 for both males and females). The gallbladders of the male overweight BMI group were also positioned significantly higher than those of the male normal BMI group (p = 0.04). No pairwise difference was found between the genders within each respective BMI group.

**Table 2:** Average Relative Vertical Translation and Log(Slope) by Gender and the BMI Group *Significant at α < 0.05; **significant at α < 0.01 BMI: body mass index

Gender	BMI group	Average relative vertical translation (mm/mm)	Average log(slope)-coronal plane (mm/mm)	Average log(slope)-sagittal plane (mm/mm)
Female	Normal	0.621	0.486	0.378
	Overweight	0.539	0.318	0.261
	Obese	0.477	0.135	0.095
Male	Normal	0.585	0.499	0.263
	Overweight	0.454	0.079	-0.093
	Obese	0.437	-0.016	-0.138
Pairwise analysis		P-value (Tamhane)	P-value (Tukey)	P-value (Tukey)
Female	Normal-overweight	0.10	0.68	1
	Overweight-obese	0.49	0.60	0.47
	Normal-obese	<0.001**	0.03*	0.25
Male	Normal-overweight	0.04*	0.004**	0.005**
	Overweight-obese	1	0.96	1
	Normal-obese	<0.001**	<0.001**	<0.001**
Between female and male	Normal-normal	0.96	1	1
	Overweight-overweight	0.23	0.29	<0.001**
	Obese-obese	0.96	0.77	0.14

Orientation

The slope of the gallbladder was calculated for the six groups to determine if the gallbladder had a more horizontal (slope → 0; log(slope) → -2) or more vertical (slope → ∞; log(slope) → 2) orientation in both the coronal and sagittal planes (Table 2). Statistically, one-way ANOVAs of log(slope) in the coronal plane (p < 0.001) and log(slope) in the sagittal plane (p < 0.001) showed that there were overall significant differences between the six groups. The gallbladders of the obese BMI groups were significantly more horizontal in the coronal plane than those of the respective gender and normal BMI group (p < 0.001 for both males and females). The gallbladders of the male overweight BMI group were also significantly more horizontal in the coronal plane than those of the male normal BMI group (p = 0.004). The orientation in the sagittal plane followed the same trend as the coronal plane between the male groups (p < 0.001 for normal versus obese and p = 0.007 for normal versus overweight) but showed no significant difference for any of the female groups. Orientation in the sagittal plane was the only dependent variable to show a significant difference between one of the gender subgroups, where the overweight males had a significantly more horizontal gallbladder in the sagittal plane than the overweight females (p < 0.001). No other pairwise difference was found between the genders within the normal and obese BMI groups in both the sagittal and coronal planes. Pearson’s correlation showed a moderate correlation (r = 0.53; p < 0.001) between the log(slope) values on the coronal and sagittal planes, indicating that the gallbladder orientation of different BMI groups is likely to show a similar pattern on both planes.

Rib cage obstruction

Obstruction by the rib cage was rated on a Likert scale based on the position of the gallbladder neck and fundus relative to the lowest point of the right costal margin (Table 3). A Kruskal-Wallis test comparing all three BMI groups for both females and males determined that at least one BMI group was statistically significantly different (females, p = 0.012; males, p = 0.002). For females, there was a significantly more rib cage obstruction of the gallbladder in obese females compared to normal females (p = 0.007). Similarly, for males, there was also significantly more rib cage obstruction in obese males compared to normal males (p < 0.001). There was no significant difference between the normal and overweight or overweight and obese groups for either gender. While there was no difference between genders within the normal and overweight BMI groups, there was significantly more rib cage obstruction in obese males than obese females (p = 0.013).

**Table 3:** Rib Cage Obstruction Likert Scale Scores by Gender and the BMI Group BMI: body mass index

Gender	BMI group	Degree of rib cage obstruction		
		0: no obstruction	1: low partial obstruction	2: high partial obstruction
Female	Normal	13	15	2
	Overweight	6	17	7
	Obese	5	19	6
Male	Normal	7	8	15
	Overweight	6	12	12
	Obese	0	10	20

## Discussion

The results from this cross-sectional study suggest that there is an association between BMI and the anatomical position of the gallbladder. The results showed that the gallbladder was positioned relatively higher and oriented more horizontally within the abdomen of individuals with obese BMI than those with normal BMI. These quantitative results support the finding that rib cage obstruction was greater in those with obese BMI compared to those with normal BMI. The results also showed that the gallbladder was positioned relatively higher and oriented more horizontally within the abdomen of individuals with overweight BMI than those with normal BMI only for males and not for females, suggesting that the association between BMI and the anatomical position of the gallbladder is more stepwise in males than females. These results may be explained by the fact that increased visceral fat on the abdomen can push abdominal organs upward within the abdomen. This would also explain why there was greater rib cage obstruction in obese males than obese females, given that males have a relatively more central abdominal adipose tissue distribution than females, who have more even distribution of adipose tissue throughout the abdominal, hip, and thigh regions [12].

This study is limited by the single point of data collection, making it difficult to derive causal relationships from this cross-sectional analysis [13]. All CT scans were performed at multiple sites of the same radiology organization, limiting the generalizability of these results. Additionally, the use of BMI as an independent variable is limited by its lack of specificity of body fat, muscle, and bone composition contributing to its numerical value. Differences in body fat or muscle composition may influence the variation in gallbladder position, which were not investigated in this study. Gallbladder pathology prevalence can also vary by race and ethnicity, which was also not analyzed in this study [14]. The results of this study may be affected by breathing and Valsalva techniques sometimes used in abdominal CT imaging, which possibly distort abdominal anatomy [15]. While this study did not investigate which breathing techniques were used and when, these breathing techniques are already adopted in the setting of ultrasound to aid in the localization of abdominal anatomy [16].

Current ultrasound protocols vary in their instruction for where to begin scanning the gallbladder, and many do not mention the possibility of taking an intercostal approach [6,16-18]. The knowledge of these anatomical considerations of the gallbladder creates an opportunity to improve the education of ultrasound novices, who may now have a more specific understanding of where to begin when performing gallbladder sonography and ultrasound-guided procedures. The study results also suggest that an intercostal approach can be recommended for gallbladder sonography in patients of higher BMI. This is beneficial because many overweight and obese patients may have less adipose in the intercostal area, increasing the feasibility of gallbladder sonography for this cohort.

## Conclusions

The purpose of this cross-sectional study was to determine if there was an association between BMI and the anatomical position of the gallbladder within the abdomen so that protocols for gallbladder sonography can be better specified. The results showed that individuals in the obese BMI group were associated with a higher, more horizontally positioned gallbladder within the abdomen that is more likely to be obstructed by the rib cage, while those in the normal BMI group had a lower, more vertically positioned gallbladder that is less obstructed by the rib cage. The knowledge of this association not only provides ultrasound novices a more targeted approach for locating the gallbladder but also provides evidence to recommend an intercostal approach for obese patients that may have not been previously candidates for gallbladder sonography.

## Appendices

Appendix A: The description of calculation for vertical translation

Two measurements were required to calculate relative vertical translation: the total distance between the midpoint of the xiphoid process and umbilicus and the vertical component of the resultant right triangle between the xiphoid process and the midpoint of the gallbladder (M). A combined vertical midpoint was calculated by averaging the values calculated from the coronal and sagittal planes (see Figure 2 for the diagram of y values): $\Delta y = \frac{(y_{1}+y_{2})}{2}$ and $\Delta y_{combined} = Average(\Delta y_{cor},\Delta y_{sag})$

A combined total distance between the xiphoid and umbilicus was calculated by averaging the values calculated from the coronal and sagittal planes: $yXU_{combined} = (y_{1}+y_{4}) or (y_{2}+y_{3})$ and $yXU_{combined} = Average[(y_{1}+y_{4})_{cor}, (y_{2}+y_{3})_{cor}, (y_{1}+y_{4})_{sag}, (y_{2}+y_{3})_{sag}]$

Vertical translation from the xiphoid process was calculated as a ratio relative to the total length of distance between the xiphoid and umbilicus to correct for variations in distance due to different patient heights.

Equation A: The Calculation of Relative Vertical Translation

Relative Vertical Translation = $\frac{\Delta y_{combined}}{\Delta yXU_{combined}}$

Appendix B: The description of calculation for orientation

Orientation was quantified to determine how horizontal or vertical the position of the gallbladder is in the coronal and sagittal planes. A straight line was drawn through the neck and fundus in the coronal or sagittal plane. The slope of this line was calculated in the coronal and sagittal planes to determine the orientation of the gallbladder in both dimensions (see Figure 2 for the diagram of x and y values).

Equation B: The Calculation of Gallbladder Slope

Slope = $(y_{1}-y_{2})/(x_{1}-x_{2})$
